# Reviewed article: Eller BBB, Junqueira MAB, Araújo LB. Congenital syphilis related to primary health care and prenatal care coverage, Minas Gerais, Brazil, 2020-2022: a spatial analysis. Epidemiol Serv Saude. 2025:34;e 20240495.

**DOI:** 10.1590/S2237-96222025v34e20240495.a

**Published:** 2025-07-11

**Authors:** Vanessa Coelho de Aquino Benjoino Ferraz

**Affiliations:** Secretaria Municipal de Saúde de Campo Grande

**Table:** 

Rating	Excellent	Good	Average	Below average	Poor
Interest	✓				
Quality		✓			
Originality		✓			
Overall		✓			

**Table:** 

Questionnaire	Yes	No	Not applicable
Does the manuscript contain new and significant information to justify publication?	✓		
Does the Abstract (Summary) clearly and accurately describe the content of the article?		✓	
Is the problem significant and concisely stated?	✓		
Are the methods described comprehensively?		✓	
Are the interpretations and conclusions justified by the results?	✓		
Is adequate reference made to other work in the field?	✓		

## Manuscript Structure

Length of article is: Adequate

Number of tables is: Adequate

Number of figures is: Too few

Please state any conflict(s) of interest that you have in relation to the review of this paper (state “none” if this is not applicable): None

## Comments

A pesquisa contribui para o SUS ao fornecer dados epidemiológicos sobre a incidência de sífilis congênita em MG de 2020 a 2022 e a relação com indicadores importantes do Previne Brasil diretamente relacionados ao diagnóstico, prevenção e tratamento precoce, o que pode auxiliar na elaboração de políticas públicas de saúde. Os resultados podem ser utilizados para embasar a alocação de recursos para programas de acompanhamento pré-natal e para a notificação dos casos.

Minhas preocupações com o artigo são:

1) Adequar o tipo de estudo do artigo pois trata-se de descrição dos achados.

2) Adequar o título ao delineamento da pesquisa e alterar o período de estudo.

3) Sugiro rever análise estatística.

4) O estudo menciona Resolução com especificações éticas de pesquisas nas ciências humanas e sociais.

5) Os métodos necessitam de mais detalhes: a) mencionar as macrorregiões de saúde que são mencionadas nos resultados e na discussão, b) incluir os sistemas de informações utilizados, c) definição detalhada da seleção dos clusters, d) não houve menção de viés no ano de 2020 devido a pandemia, por conseguinte baixa incidência de casos e demais indicadores.

6) As ilustrações precisam ser revisadas: a) presença de numeração igual, b) sugiro mapas diferentes para cada ano c) a tabela 1 traz o valor de p na linha e deve-se colocar na coluna ou suprimir por todos serem significativos e inserir no texto. d) a segunda figura 1 traz siglas em inglês e deve-se adequar para o português. e) é necessária adequação nos eixos para ficar com tamanho mais adequado. f) adequar as legendas e cores para a possibilidade de impressão preto e branco ficar compreensível e de alta qualidade.

Verificar a palavra-chave “sífilis em gestantes “ que não está contemplada no DeCS.

7) O manuscrito precisa ser revisado para corrigir o uso de maiúsculas, como “Previne Brasil”, “Organização Mundial de Saúde”, Ministério da Saúde”.

